# Prof. Edward M. Moore

**Published:** 1854-09

**Authors:** 


*Prof. Edward M. Moore.*—It will be seen from a notice on another page, that this gentleman has found it necessary to resign the Chair of Anatomy in the Buffalo Medical College, in consequence of his removal to Columbus, Ohio, to take the professorship of Surgery in the Starling Medical College.

In this separation Prof. Moore's colleagues, of the Buffalo school, feel unusual regret. His popularity as a teacher was such as to make him a valuable addition to the force of the school, while his character as a man rendered him a very pleasant associate to his colleagues.

We hope that in this transfer of his interests from New York to Ohio, the Professor may realize his highest anticipations. H.

